# A feasibility and acceptability study of screening the parents/guardians of pediatric dental patients for the social determinants of health

**DOI:** 10.1186/s40814-023-01269-3

**Published:** 2023-03-09

**Authors:** Raghbir Kaur, Martin Lieberman, Margaret K. Mason, Isaac P. Dapkins, Rebecca Gallager, Kathleen Hopkins, Yinxiang Wu, Andrea B. Troxel, Ayah Rashwan, Chelsea Hope, Daniel J. Kane, Mary E. Northridge

**Affiliations:** 1 Department of Dental Medicine, Advanced Education in Pediatric Dentistry Program, Family Health Centers at NYU Langone, 150 55Th Street, First Floor, Brooklyn, NY 11220 USA; 2 Graduate Dental Education and Distance Learning, NYU Langone Dental Medicine Postdoctoral Residency Programs, Family Health Centers at NYU Langone, Department of Dental Medicine, Hansjörg Wyss Department of Plastic Surgery, NYU Grossman School of Medicine, 5800 Third Avenue, Third Floor, Brooklyn, NY 11220 USA; 3 Department of Dental Medicine, Family Health Centers at NYU Langone, 5800 Third Avenue, Room 320, Brooklyn, NY 11220 USA; 4 Departments of Medicine and Population Health, Family Health Centers at NYU Langone, NYU Grossman School of Medicine, 5800 Third Avenue, Suite 2-020, Brooklyn, NY 11220 USA; 5 Youth and Adolescent Services, Family Health Centers at NYU Langone, 150 55Th Street, Brooklyn, NY 11256 USA; 6 Department of Community Programs, Family Health Centers at NYU Langone, 6025 6Th Avenue, Second Floor, Brooklyn, NY 11220 USA; 7 Department of Population Health, NYU Grossman School of Medicine, 180 Madison Avenue, Fifth Floor, New York, NY 10016 USA; 8 Division of Biostatistics, Department of Population Health, NYU Grossman School of Medicine, 180 Madison Avenue, Fifth Floor, 5-55, New York, NY 10016 USA; 9 Department of Dental Medicine, Advanced Education in Pediatric Dentistry Program, Family Health Centers at NYU Langone, 150 55Th Street, Third Floor, Brooklyn, NY 11220 USA; 10 Family Health Centers at NYU Langone, Department of Dental Medicine, Hansjörg Wyss Department of Plastic Surgery, NYU Grossman School of Medicine, 5800 Third Avenue, Room 344, Brooklyn, NY 11220 USA

**Keywords:** Community health centers, Community resources, Delivery of health care, Federally Qualified Health Center, Health equity, Interprofessional education, Pediatric dentistry, Quality of health care, Screening, Social determinants of health (10; limit = 10)

## Abstract

**Background:**

The social determinants of health (SDOH) are the conditions in which people are born, grow, work, live, and age. Lack of SDOH training of dental providers on SDOH may result in suboptimal care provided to pediatric dental patients and their families. The purpose of this pilot study is to report the feasibility and acceptability of SDOH screening and referral by pediatric dentistry residents and faculty in the dental clinics of Family Health Centers at NYU Langone (FHC), a Federally Qualified Health Center (FQHC) network in Brooklyn, NY, USA.

**Methods:**

Guided by the *Implementation Outcomes Framework*, 15 pediatric dentists and 40 pediatric dental patient–parent/guardian dyads who visited FHC in 2020–2021 for recall or treatment appointments participated in this study. The a priori feasibility and acceptability criteria for these outcomes were that after completing the *Parent Adversity Scale* (a validated SDOH screening tool), ≥ 80% of the participating parents/guardians would feel comfortable completing SDOH screening and referral at the dental clinic (acceptable), and ≥ 80% of the participating parents/guardians who endorsed SDOH needs would be successfully referred to an assigned counselor at the Family Support Center (feasible).

**Results:**

The most prevalent SDOH needs endorsed were worried within the past year that food would run out before had money to buy more (45.0%) and would like classes to learn English, read better, or obtain a high school degree (45.0%). Post-intervention, 83.9% of the participating parents/guardians who expressed an SDOH need were successfully referred to an assigned counselor at the Family Support Center for follow-up, and 95.0% of the participating parents/guardians felt comfortable completing the questionnaire at the dental clinic, surpassing the a priori feasibility and acceptability criteria, respectively. Furthermore, while most (80.0%) of the participating dental providers reported being trained in SDOH, only one-third (33.3%) usually or always assess SDOH for their pediatric dental patients, and most (53.8%) felt minimally comfortable discussing challenges faced by pediatric dental patient families and referring patients to resources in the community.

**Conclusions:**

This study provides novel evidence of the feasibility and acceptability of SDOH screening and referral by dentists in the pediatric dental clinics of an FQHC network.

## Key messages regarding feasibility

1. Uncertainties existed regarding the feasibility and acceptability of social determinants of health screening and referral by dentists in the pediatric dental clinics of a health center.
2. The key feasibility finding is that 83.9% of the participating parents/guardians who expressed a social determinants of health need were successfully referred to a counselor for follow-up.
3. The implications of the feasibility findings for the design of the main study are that more interprofessional training is needed for dental residents at the health center to feel comfortable discussing challenges faced by patient families and referring them to resources in the community.

## Background

The World Health Organization defines the social determinants of health (SDOH) as the nonmedical factors that influence health outcomes, commonly referred to as the conditions in which people are born, grow, work, live, and age [1]. Government reports [2–4] and peer-reviewed publications [5–11] have underscored the disproportionate burden of poor oral health on people across the life course who are impoverished and/or members of racial/ethnic minority or immigrant populations who experience suboptimal access to quality oral health care.

Despite being preventable, dental caries and gingival and periodontal diseases are the most prevalent oral diseases of childhood, with associated pain, infection, and loss of function that exhibit a social gradient, thus linking the SDOH to oral health [12–16]. Lack of training of dental providers on SDOH may result in suboptimal care resulting from unconscious bias, incomplete or inaccurate information, treatments that are difficult for patients and their caregivers to follow, and distrustful interactions among patients, providers, and family members [17]. Accordingly, the Commission on Dental Accreditation (CODA) recently implemented the Accreditation Standards for Advanced Specialty Education Programs in Pediatric Dentistry to include both didactic instruction on the impact of SDOH on oral and general health and clinical experiences that enable resident competency in interprofessional communication and collaborative care [18]. Systematically screening and referring for SDOH during primary care may help identify environmental and social factors affecting children’s health and lead to the receipt of more community resources for families [19, 20]. The most common SDOH domains screened for in children include the family context and economic stability, usually followed by referrals and/or interventions to address identified SDOHs [21]. Furthermore, greater use of the Centers for Medicare and Medicaid Services (CMS) Z codes associated with SDOH during patient encounters has been reported over time, notably for homelessness, which may lead to improved care coordination and identify opportunities to advance health equity [22, 23].

Federally Qualified Health Centers (FQHCs) were founded over 55 years ago to target the roots of poverty by combining the resources of local communities with federal funds to establish neighborhood clinics in underserved areas [24]. At Family Health Centers at NYU Langone (FHC), an FQHC network in Brooklyn, NY, USA, with multiple sites located in largely immigrant and impoverished neighborhoods, medical providers screen families for SDOH at annual visits and provide referrals when warranted to facilitate access to available resources and services. Since pediatric dentists often interact with patients multiple times per year, screening for SDOH at dental visits may augment that provided at medical visits and better ensure that families gain the assistance they need. Moreover, training pediatric dental residents to work closely with medical, behavioral health, and social service providers to screen and refer families for SDOH needs may enhance resident competency in interprofessional communication and collaborative care [18]. Therefore, the objectives of this study are to report the feasibility and acceptability of SDOH screening and referral in the dental setting.

## Methods

This research is guided by the *Implementation Outcomes Framework*, which posits four nested levels of change for assessing performance improvement (individual, group/team, organization, and larger system/environment) and encompasses implementation, service, and client outcomes [25]. The two implementation outcomes of central interest in this study are feasibility and acceptability [26]. Feasibility is defined as the extent to which an innovation can be successfully used or carried out within a given agency or setting [27], which in the case of this study is SDOH screening and referral in the dental clinics of an FQHC network. Acceptability is the perception among implementation stakeholders—in the case of this study, the parents/guardians of pediatric dental patients—which a given innovation is agreeable, palatable, or satisfactory [26].

### Approach and participants for the study

The NYU Grossman School of Medicine Institutional Review Board (IRB) reviewed and approved all study procedures (s20-00,696) for this prospective mixed-methods study. The IRB-approved protocol that includes the study instruments is available from the corresponding author upon written request. All Health Insurance Portability and Accountability Act (HIPAA) safeguards were followed. The provider participants in the study consist of 15 pediatric dentists (residents and faculty) employed at FHC who were introduced to the study during departmental meetings held in 2020, expressed a desire to be involved, provided informed consent, and underwent calibration on the administration of the SDOH screening instrument to the parents/guardians and the referral process for those desiring follow-up. The patient and parent/guardian participants consist of 40 pediatric dental patient–parent/guardian dyads who visited FHC in 2020–2021 for regularly scheduled dental appointments, were introduced to the study by a research team dentist who was not involved in treating the pediatric dental patient, and provided informed consent.

Feasibility was assessed using pediatric dental provider surveys administered pre- and post-implementation of the SDOH screening and referral protocol and the case management log of the assigned counselor in the Family Support Center at the health center. The a priori feasibility criterion was that post-intervention, 80% or more of the parents/guardians who endorsed SDOH needs were successfully referred to an assigned counselor at the Family Support Center. Acceptability was assessed using a parental/guardian perception questionnaire administered after completion of the SDOH screening and referral protocol. The a priori acceptability criterion was that 80% or more of the parent/guardian participants would feel comfortable completing the SDOH screening and referral intervention at the dental clinic.

### Data collection methods and instruments

The demographic characteristics of the pediatric dental patient and parent/guardian participants were obtained from the Dentrix electronic health record (EHR) for each patient and supplemented by direct report of the parent/guardian participants prior to their completion of the following two surveys administered in English and Spanish: (1) *Parent Adversity Scale*, with validated questions pertaining to food security, basic household needs, literacy, personal safety, transportation needs, and other domains based on findings from the Institute of Medicine and CMS [19], and (2) *Parent/Guardian Perceptions of Adversity Screening at Dental Visits*, with questions on the acceptability of the SDOH screening and referral intervention in dental clinics that was developed by the principal investigator (R. K.) for this study. If they so desired, the parents/guardians who identified SDOH needs were then referred to the Family Support Center with case management provided by an assigned counselor who assisted them in obtaining required referrals, resources, and services.

The dental provider participants completed both pre- and post-intervention surveys developed by the principal investigator (R. K.) for this study regarding their experiences screening patients and their families for SDOH and their comfort in doing so on an ordinal rating scale (minimally comfortable, moderately knowledgeable, competent, highly experienced). Moreover, the pre-intervention survey also included questions on provider status (resident, faculty), years practicing dentistry, and training in SDOH. Finally, the post-intervention survey also included open-ended questions seeking suggestions for improving resident training in SDOH and recommendations for enhancing the intervention protocol.

### Data analysis

For descriptive statistics, continuous variables were summarized with means, standard deviations (SD), and minimums-maximums for normally distributed variables and medians and interquartile ranges (IQR) for non-normally distributed variables; categorical variables were summarized with counts and percentages. Identified SDOH needs and number of SDOH needs endorsed by the participating parents/guardians were calculated overall and by the age group of the pediatric patients ($\le$ 7 years vs. > 7 years), with the cut point chosen as the mean rounded to the closest integer of the pediatric patient age distribution.

Analysis of the open-ended question answers was conducted using thematic content analysis [28, 29]. To enhance the validity of the coding scheme, multiple members of the study team began the qualitative data analysis by each independently reading all feedback on the open-ended questions to understand in greater depth the dental provider responses regarding their perceptions of the intervention. Next, relevant quotations were organized by each possible response. As a final step, quotations were selected for inclusion that best represents the perceptions described by the provider participants.

## Results

The demographic characteristics of the participating pediatric dental patients and their parents/guardians are provided in Table 1.

**Table 1:** Demographic characteristics of the participating pediatric dental patients (*n* = 40) and their parents/guardians (*n* = 40)

Characteristic	Descriptive statistics^a^
Age of pediatric dental patient, y	
Mean (SD)	7.3 (3.0)
Minimum–maximum	2–12
Age range of pediatric dental patient, y^b^	
Toddler (2–3)	4 (10.0)
3–5	10 (25.0)
Middle childhood (6–8)	12 (30.0)
Middle childhood (9–11)	9 (22.5)
Young teen (12–14)	5 (12.5)
Language spoken at home for pediatric dental patient	
English	19 (47.5)
Spanish	21 (52.5)
Dental insurance of pediatric dental patient	
Private	1 (2.5)
Medicaid	38 (95.0)
Child Health Plus (New York State of Health Marketplace)	1 (2.5)
Age of parent/guardian, y	
Mean (SD)	36.5 (8.7)
Minimum–maximum	21–68
Age range of parent/guardian, y	
19–25	2 (5.0)
26–35	19 (47.5)
36–45	14 (35.0)
46–55	4 (10.0)
56 +	1 (2.5)
Relationship of parent/guardian to pediatric dental patient	
Mother	35 (87.5)
Father	4 (10.0)
Guardian	1 (2.5)
Marital status of parent/guardian	
Married	17 (42.5)
Single	23 (57.5)
Race/ethnicity of parent/guardian	
Hispanic	26 (65.0)
Asian	1 (2.5)
Black	5 (12.5)
White	8 (20.0)

^a^Mean (standard deviation = SD) and minimum–maximum are presented for continuous variables; *n* (%) are presented for categorical variables

^b^Centers for Disease Control and Prevention child development age ranges available at https://www.cdc.gov/ncbddd/childdevelopment/positiveparenting/index.html

The pediatric dental patients in this study ranged in age from 2 to 12 years with a mean of 7.3 years (*SD* = 3.0), and their parents/guardians ranged in age from 21 to 68 years with a mean of 36.5 years (*SD* = 8.7). Most of the participating parents/guardians were the mothers of the pediatric dental patients (87.5%), most reported being of single marital status (57.5%), and most were of Hispanic race/ethnicity (65.0%).

The types and number of SDOH needs endorsed by the participating parents/guardians overall and by the age group of the pediatric patients ($\le$ 7 years vs. > 7 years) are provided in Table 2.

**Table 2:** Types and number of needs endorsed by the parents/guardians of pediatric dental patients on the *Parent Adversity Scale*, overall, and by the age group of the pediatric patient ($\le$ 7 years vs. > 7 years)

	**Overall**	**Pediatric patient **$\le$** 7 years**	**Pediatric patient > 7 years**
*n*	40	21	19
**Needs identified by parents/guardians**	***n* (%)**	***n* (%)**	***n* (%)**
Within the past year, worried that food would run out before had money to buy more	18 (45.0)	6 (28.6)	12 (63.2)
Within the past year, there was a time that bought food that did not last and did not have money to buy more	16 (40.0)	7 (33.3)	9 (47.4)
Would like classes to learn English, read better, or obtain high school degree	18 (45.0)	7 (33.3)	11 (57.9)
Need help getting things for child (diapers, car seats, cribs, strollers)	7 (17.5)	5 (23.8)	2 (10.5)
Need help finding someone to watch child while working and taking classes	9 (22.5)	3 (14.3)	6 (31.6)
Have problems with home (mold, broken walls, peeling paint, pests)	3 (7.5)	2 (9.5)	1 (5.3)
Worry that family will not have a place to live (cannot pay rent, electricity will be turned off, eviction)	7 (17.5)	3 (14.3)	4 (21.1)
Feel unsafe at home due to domestic violence	1 (2.5)	1 (4.8)	0 (0.0)
Need help signing up for programs to help family find health insurance or pay less taxes	9 (22.5)	4 (19.0)	5 (26.3)
Need help from a lawyer (immigration status, divorce, custody)	5 (12.5)	2 (9.5)	3 (15.8)
Worried about child’s behavior (tantrums, hitting)	2 (5.0)	1 (4.8)	1 (5.3)
In past 12 months, lack of reliable transportation kept from medical appointments, meetings, work, and getting things	7 (17.5)	3 (14.3)	4 (21.1)
Need help for self or someone in household cutting down on smoking, drinking, and drug use	0 (0.0)	0 (0.0)	0 (0.0)
**Number of needs endorsed**			
Multiple needs	24 (60.0)	9 (42.9)	15 (78.9)
Single need	7 (17.5)	4 (19.0)	3 (15.8)
No needs	9 (22.5)	8 (38.1)	1 (5.3)
Number of needs (median [inter-quartile range])	2 [1, 4]	1 [0, 4]	3 [2, 4]

The most prevalent SDOH needs endorsed by parents/guardians were worried within the past year that food would run out before had money to buy more and would like classes to learn English, read better, or obtain a high school degree (both at 45.0% overall), followed closely by a time within the past year that bought food that did not last and did not have money to buy more (40.0% overall). All three of these endorsed SDOH needs were more prevalent in the parents/guardians of the older (> 7 years) vs. younger ($\le$ 7 years) patients.

Regarding the total number of SDOH needs endorsed by the participating parents/guardians, 38.1% endorsed no needs, and 42.9% endorsed multiple needs in the younger pediatric patient age group, whereas 5.3% endorsed no needs and 78.9% endorsed multiple needs in the older pediatric patient age group. When the number of SDOH needs endorsed was analyzed as a numerical variable, the median [IQR] number of SDOH needs in the overall sample was 2 [1, 4], with 1 [0, 4] for the younger pediatric patient age group and 3 [2, 4] for the older pediatric patient age group.

The parent/guardian responses/perceptions following completion of the *Parent Adversity Scale* are presented in Table 3.

**Table 3:** Parent/guardian responses/perceptions following completion of the *Parent Adversity Scale* (*n* = 40)

Item	Response = *n* (%)	Illustrative quotation of parent/guardian perceptions
Previously completed similar questionnaire?	Yes = 10 (25.0)	*Yes, similar but not exact same*
	No = 28 (70.0)	*No, this is the first time*
	Do not remember = 2 (5.0)	*I don't remember*
If yes (*n* = 10), what happened afterward?	No follow-up = 5 (50.0)	*Nothing happened. No one got in contact with me*
	No need for services at the time = 3 (30.0)	*Mother did not need services at the time*
	Unable to access services offered = 2 (20.0)	*They asked if she wanted to learn English and set her up with someone. However, classes were in the daytime when her children were home. She asked if she could do it at night but they could not*
Comfortable completing questionnaire at dental clinic?	Yes = 38 (95.0)	*Yes. I feel comfortable. Its a bad year for everyone, and I lost my job and income*
	No = 2 (5.0)	*Not exactly because I don't know what this is about. She filled out a form like this and never heard more about it*
Prefer answering questions at dental clinic or medical clinic and why?	Dental = 12 (30.0)	*The communication is better. Maybe you have more time. You are knowledgeable and educated. You are kind, and my family could use some help with transportation services*
	Medical = 2 (5.0)	*The medical clinic is better because it is closer to my home so I get there better. It is more accessible*
	No difference/unclear = 26 (65.0)	*They are the same at the dentist and pediatrician. The questions are asked properly in Spanish*
Can dentist serve as resource to help get needed support?	Yes = 36 (90.0)	*I think yes. This place they really do try to help you. Doctors here go above and beyond to help. So yes, I believe this will help*
	No/unsure = 4 (10.0)	*I would assume so. It would be helpful; my only concern if there is a language barrier. Would be helpful to have this in other languages*

Most (95.0%) parent/guardian participants felt comfortable completing the questionnaire at the dental clinic, surpassing the a priori acceptability criterion of 80% or more. Nearly two-thirds (65.0%) of the parents/guardians endorsed no preference for answering the SDOH questions at the dental clinic or medical clinic. Finally, 90.0% of the parents/guardians believed that their dentist could serve as a resource to help them get the support they and their family need.

The pre-implementation survey results for the pediatric dental provider participants are presented in Table 4.

**Table 4:** Pediatric dental provider pre-implementation survey for screening parents/guardians of pediatric patients for social determinants of health needs using the Parent Adversity Scale (*n* = 15)

Item	Descriptive statistics^a^
Dental provider position	
Resident	7 (46.7)
Faculty	8 (53.3)
If resident (*n* = 7), what year?	
Postgraduate year 1 (PGY1)	4 (57.1)
Postgraduate year 2 (PGY2)	3 (42.9)
Years practicing dentistry	3 (1, 16)
Trained in social determinants of oral health (SDOH)	
Yes	12 (80.0)
No	3 (20.0)
If trained in SDOH (*n* = 12), how was training provided?^b^	
Academic lectures in dental school curriculum	2 (16.7)
Academic lectures in residency curriculum	7 (58.3)
Continuing education courses	4 (33.3)
None of the listed choices	1 (8.3)
Residency training prepared me to screen for SDOH in pediatric dental patients^c^	
Agree	8 (61.5)
Disagree	5 (38.5)
How often do you assess SDOH for pediatric dental patients?	
Never	2 (13.3)
Almost never (less than 25% visits)	4 (26.7)
Sometimes (25% to less than 49% visits)	4 (26.7)
Usually (50% to less than 75% visits)	3 (20.0)
Almost always (more than 75% visits)	2 (13.3)
Screen pediatric dental patients for the following needs	
Child development	5 (33.3)
Culture	4 (26.7)
Family function	1 (6.7)
Food insecurity	3 (20.0)
Healthcare system characteristics	3 (20.0)
Language barrier	9 (60.0)
Physical environment	2 (13.3)
Physical safety	2 (13.3)
Social capital	0 (0.0)
Social support	1 (6.7)
Socioeconomic status	4 (26.7)
Substance abuse	3 (20.0)
Use of dental care	9 (60.0)
How competent feel discussing challenges faced by pediatric dental patient families	
Minimally comfortable	7 (46.7)
Moderately knowledgeable	7 (46.7)
Competent	1 (6.7)
How competent feel referring patients to available resources in the community	
Minimally comfortable	7 (46.7)
Moderately knowledgeable	7 (46.7)
Competent	1 (6.7)

^a^*n* (%) are presented for categorical variables, median (interquartile range) for continuous variables

^b^Certain participants endorsed more than one training

^c^Missing values account for differences in the denominators

The 15 provider participants who completed the pre-implementation survey were approximately evenly split between residents (46.7%) and faculty (53.3%), and most (80.0%) were trained in SDOH. A single provider participant (6.7%) felt confident discussing the challenges faced by pediatric dental patient families and referring patients to available resources in the community.

The post-implementation survey results for the pediatric dental provider participants are provided in Table 5.

**Table 5:** Pediatric dental provider post-implementation survey for screening parents/guardians of pediatric patients for social determinants of health (SDOH) needs using the *Parent Adversity Scale* (*n* = 13)

Item	Response = *n* (%)	Illustrative quotation of dental provider perceptions
How comfortable feel asking patients to complete the screening questionnaire	Moderately knowledgeable = 6 (46.2)	*I didn’t know how to word things because it is out of my comfort zone*
	Comfortable = 7 (53.8)	*My experience has been good because the parents were receptive to filling it out and they had some understanding of why the survey was being conducted so they were generally receptive*
Need more training in SDOH and screening for patient needs?	Yes = 8 (61.5)	*Yes, definitely. The patients coming to us by default need help so learning more would benefit us as providers*
	No = 5 (38.5)	*No, because I think it all deals with sensitivity. As a pediatric dentist, you need to be compassionate and not judgmental*
Will this screening questionnaire benefit our patients?	Yes = 13 (100.0)	*Yes, I feel like without something like this, we don’t understand why they might be cancelling an appointment or then the families aren’t aware of free resources that are available*
How competent feel discussing challenges faced by pediatric dental patient families	Minimally comfortable = 7 (53.8)	*Minimally comfortable, because it is such a sensitive topic. I’m very private about money, and I think that these questions all come back to money*
	Moderately knowledgeable = 2 (15.4)	*Moderately knowledgeable. I think being used to the verbiage of the questions would be beneficial since these are hard topics*
	Competent = 4 (30.8)	*I feel competent. I had a patient discuss domestic abuse with me because I was joking with their child and the mother opened up to me. I would feel obligated and comfortable to discuss these issues with families*
How competent feel referring patients to available resources in the community	Minimally comfortable = 7 (53.8)	*Minimally comfortable. I don’t feel very comfortable talking to them about it before. It’s kind of awaked me because we haven’t been trained to talk to them about stuff like that. It would be nice to get training on how to talk, especially if we are going to be offering help*
	Moderately knowledgeable = 2 (15.4)	*Moderately knowledgeable. I would like to understand how the referral process works because I’m unsure. I would also feel more competent if I followed up with the parents, and they explained to me the process they went through. It would make me feel more experienced*
	Competent = 4 (30.8)	*Competent. I’m sensitive to everyone’s needs. As a practitioner, listen to your patient. That’s why when you listen, these patients will volunteer information to you. I would like to see continuity*

All 13 (100%) of the provider participants who completed the post-implementation survey reported that screening and referring patient families for SDOH needs in the dental setting are of benefit to patients. Four (30.8%) provider participants felt confident either discussing the challenges faced by pediatric dental patient families or referring patients to available resources in the community vs. only one (6.7%) in the pre-implementation survey.

At the time of this writing, 26 of the 31 participating parents/guardians (83.9%) who expressed an SDOH need were successfully referred to an assigned counselor at the Family Support Center for follow-up, surpassing the a priori feasibility criterion of 80%. Of these 26 parents/guardians, 17 (65.4%) speak Spanish, and 9 (34.6%) speak English as their preferred language. Five (19.2%) parents/guardians have not yet spoken with an assigned counselor despite being called and left messages, and 1 (3.8%) had an invalid phone number. Of the 20 parents/guardians who spoke with an assigned counselor, 4 (20.0%) declined the offered services, 1 (5.0%) was already a client of the Family Support Center prior to the study, and 15 (75.0%) new clients received needed SDOH support. Referrals, resources, and services provided to date include the following: referrals to English as a Second Language (ESL) classes (*n* = 9); food pantry appointments (*n* = 7); assistance in applying for the Supplemental Nutrition Assistance Program (SNAP) (*n* = 4); provision of school supplies for children (*n* = 2); diaper and baby clothes pickup (*n* = 1); referral to Church Avenue Merchant Block Association (CAMBA), a Brooklyn-based nonprofit organization (*n* = 1); referral to New York City (NYC) Department of Homeless Services (DHS) Prevention Assistance and Temporary Housing (PATH) shelter (*n* = 1); referral to housing resources (*n* = 1); referral to NYC311 to report problems (*n* = 1); referral to NYC Care, a healthcare access program that guarantees low-cost and no-cost services to New Yorkers who do not qualify for or cannot afford health insurance, with services provided through NYC Health + Hospitals (*n* = 1); and referral to the Family Support Center office of health insurance (*n* = 1).

## Discussion

This is the first study of which we are aware in the scientific literature to report the feasibility and acceptability of SDOH screening and referral in a pediatric dental clinic of an FQHC network. Most research conducted to date on the topic of SDOH screening and referral has been in primary care, including reports related to SDOH screening tools, domains, processes, and guides for clinicians [21, 30–33].

The current feasibility and acceptability study was conducted within the initial year of the COVID-19 pandemic in the USA (summer 2020–spring 2021), with severe health, social, and economic impacts for the largely immigrant and impoverished communities in the catchment area of FHC in Brooklyn, NY, USA. Working closely with the Family Support Center leadership and staff (K. H. and R. G.) and the chief medical officer (I. P. D.) of the health center who had implemented SDOH screening and referral in the medical clinics at annual visits, the Advanced Education in Pediatric Dentistry Program within the Department of Dental Medicine sought to ensure that SDOH detection by dental residents and faculty was part of a comprehensive, integrated approach that respected the needs, priorities, and autonomy of pediatric dental patients and their families [34]. There were accordingly few refusals to participate among the parents/guardians of pediatric patients or their dental providers.

The objectives of this study were met, in that SDOH screening and referral in the dental setting were found to be both feasible and acceptable. Specifically, 83.9% of the participating parents/guardians who expressed an SDOH need were successfully referred to an assigned counselor at the Family Support Center for follow-up, exceeding the a priori feasibility criterion of 80%. In addition, 95.0% of the participating parents/guardians felt comfortable completing the questionnaire at the dental clinic, surpassing the a priori acceptability criterion of 80%. The acceptability result is consistent with the finding that the caregivers of hospitalized pediatric patients had favorable opinions of physician screening for SDOH [35].

As is true of any screening program, SDOH screening and referral should only be undertaken if there are potential benefits to those who are screened, and this feasibility and acceptability study met that mark. Situated within an FQHC network with a range of medical, dental, behavioral health, and social services along with a strong network of community partners, follow-up of families and linkage to resources were made possible through an assigned counselor at the Family Support Center for participating families who accepted available SDOH support (*n* = 15). The most frequent referrals, resources, and services provided to date for these 15 families were referrals to ESL classes (60.0%), food pantry appointments (46.7%), and assistance in applying for SNAP (26.7%).

Nonetheless, it is worth emphasizing that not all parents/guardians screened endorsed SDOH needs or accepted available support. Specifically, none of the parents/guardians screened reported needing help for themselves or someone in their household on cutting down on smoking, drinking, or drug use, and only 1 participating parent/guardian (2.5%) reported feeling unsafe at home due to domestic violence. Even for those who endorsed SDOH needs, not all screened families accepted the offered referrals, services, or resources, perhaps because they had access to alternate sources of support or because of the administrative burdens associated with social welfare policies and programs that appear to be more focused on excluding ineligible individuals than on including eligible individuals [36].

Instead of being viewed as a simple “screen-and-refer” procedure, SDOH screening and referral at chairside ought to be considered as an opportunity to initiate a discussion with families to solicit their priorities and concerns [34]. A trusted relationship with a dentist, encompassed in the philosophy of a dental home, may lead to more accessible, family-centered, continuous, comprehensive, coordinated, compassionate, and culturally competent care [37].

Findings from this feasibility and acceptability study suggest that dentists may be part of a comprehensive, integrated approach to early SDOH detection, referral, and linkage, as 90.0% of participating parents/guardians believe that dentists can serve as resources to gain needed support. This has implications for dental education and training. While most (80.0%) of the participating dental providers reported being trained in SDOH, only one-third (33.3%) usually or always assess SDOH for their pediatric dental patients. Post-implementation, all 13 (100.0%) of the participating dental providers believed that the SDOH screening questionnaire would benefit patients, but most (53.8%) felt minimally comfortable discussing challenges faced by pediatric dental patient families and referring patients to resources in the community.

The COVID-19 pandemic has tested oral healthcare providers as never before [3]. To maximize access to quality, coordinated care for underserved children and their families, integration, and workforce expansion efforts must support members of the dental team to work at the top of their collective scope of licensing capabilities [3].

This feasibility and acceptability study was focused on the individual level of the *Implementation Outcomes Framework*, specifically on dental providers and parents/guardians, where knowledge, skill, and expertise are key to change [25]. Next steps are to work at the group/team level (where cooperation, coordination, and shared knowledge are key to change), which will necessarily involve champions and implementation leaders across departments working on SDOH detection, referral, and linkage, all in the context of respecting the needs, priorities, and autonomy of patients and their families [25, 34]. This will also entail selecting, developing, testing, and evaluating additional implementation outcomes (such as costs), service outcomes (such as patient centeredness), and client outcomes (such as satisfaction) as part of the broader implementation of SDOH screening and referral in the 6 FHC dental clinics [26].

With funding from the Health Resources and Services Administration (HRSA) and in partnership with the NYU School of Global Public Health, the NYU Langone Dental Medicine Postdoctoral Residency Programs recently created five new public health courses that are currently available to all residents and faculty across programs on its virtual education platform, namely: (1) *Social Determinants of Oral Health: Science and Clinical Implications* (taught by R. K.), (2) *Substance Use & Dental Health: A Public Health Perspective*, (3) *Mental & Oral Health: A Public Health Perspective*, (4) *Sugary Beverages and Public Health Dentistry*, and (5) *Special Care Dentistry*. Introducing clinical training for pediatric dental residents in the Family Support Center and having faculty guide them in practicing motivational interviewing (MI) techniques with their pediatric patients [38] would be of value in enhancing resident confidence in discussing SDOH challenges faced by patients and their families and resident familiarity with available medical, behavioral health, and social services in the health center and SDOH resources in the community. Note that an educational intervention for medical interns consisting of both a didactic and an experiential component that involved shadowing social workers increased their comfort and knowledge of SDOH and community resources as well as their documentation of social questions [39].

The limitations of this feasibility and acceptability study include the small sample size of participating dental providers, which precluded testing of statistically significant differences pre- and post-implementation regarding their perceived competency in SDOH screening and referral. Furthermore, this feasibility and acceptability study was conducted in a single FQHC network during the initial year of the COVID-19 pandemic in Brooklyn, NY, USA, and may not be generalizable to other FQHCs in diverse locales or alternate time periods. Instead, adaptations may be necessary to meet the needs of local populations and communities. Finally, the pre- and post-surveys developed for the dental providers for this study would benefit from distinguishing among the concepts of comfort, knowledge, competence, and experience.

Nonetheless, this feasibility and acceptability study adds to the evidence base underscoring the importance of educating pediatric dental residents and faculty on SDOH and enhancing their clinical experiences in interprofessional communication and collaborative care. The implementation of a new electronic health record (EHR) titled Epic with Wisdom in November 2021 at NYU Langone Health that integrates dental care with other services provided at FHC may assist with SDOH tracking of pediatric dental patients and their parents/guardians and the receipt of desired SDOH resources by these families. This represents an SDOH intervention at the organization level of the *Implementation Outcomes Framework*, where structure and strategy are key to change [25].

While this feasibility and acceptability study is part of a nascent movement toward integration of oral, medical, and behavioral health with social services within an FQHC network, interventions at the larger system/environment level (where reimbursement, legal, and regulatory policies are key to change) are critical to address the ongoing economic distress compounded by the COVID-19 pandemic and its long-term health impacts for disadvantaged patients and communities [3, 26, 36]. Along with recognized system barriers such as time constraints and the greater value placed on medical vs. social knowledge in training programs [40], administrative burdens associated with social welfare policies and programs include complicated eligibility requirements, burdensome documentation, and the stigma and discrimination involved in bureaucratic interactions [36].

Future implementation research that incorporates the views of individuals involved at every step of the SDOH process may yield ideas for improving both the training of residents and faculty and the lives of their patients and family members. Attention needs to be paid not only to the design of social and economic policies but also to their administration [36]. In the final analysis, if the administration of a program limits access to health-promoting policies, it undermines the health and well-being of the people it is intended to serve.

## Conclusions

Findings from this feasibility and acceptability study suggest that dentists may be part of a comprehensive, integrated approach to early SDOH detection, referral, and linkage. Concerted efforts to educate FHC pediatric dental residents and faculty on SDOH and enhance their clinical experiences in interprofessional communication and collaborative care are ongoing to increase their confidence in discussing challenges faced by patients and their families and referring them to services and resources. Future implementation research with in-depth qualitative interviews of individuals involved at every step of the process (family members, patients, dental faculty and residents, administrators, and staff) is needed to better ensure SDOH screening and referral clinical workflows are optimized to support patients and their families in need of social services.

## Data Availability

De-identified raw data and materials described in the manuscript are freely available from the corresponding author on reasonable request.

## Abbreviations

CAMBA: Church Avenue Merchant Block Association
CMS: Centers for Medicare and Medicaid Services
CODA: Commission on Dental Accreditation
DHHS: Department of Health and Human Services
DHS: Department of Homeless Services
EHR: Electronic health record
ESL: English as a second language
FHC: Family Health Centers at NYU Langone
FQHC: Federally Qualified Health Center
HIPAA: Health Insurance Portability and Accountability Act
HRSA: Health Resources and Services Administration
IRB: Institutional review board
MI: Motivational interviewing
NYC: New York City
NYU: New York University
PATH: Prevention Assistance and Temporary Housing
SD: Standard deviation
SDOH: Social Determinants of Health
SNAP: Supplemental Nutrition Assistance Program
US: United States
